# Mechanical Structural Design of a Piezoresistive Pressure Sensor for Low-Pressure Measurement: A Computational Analysis by Increases in the Sensor Sensitivity

**DOI:** 10.3390/s18072023

**Published:** 2018-06-24

**Authors:** Anh Vang Tran, Xianmin Zhang, Benliang Zhu

**Affiliations:** Guangdong Key Laboratory of Precision Equipment and Manufacturing Technology, School of Mechanical and Automotive Engineering, South China University of Technology, Guangzhou 510640, China; vangtrananh@gmail.com (A.V.T.); zhangxm@scut.edu.cn (X.Z.)

**Keywords:** MEMS, piezoresistive pressure sensor, finite element method, sensitivity

## Abstract

This paper proposes a novel micro-electromechanical system (MEMS) piezoresistive pressure sensor with a four-petal membrane combined with narrow beams and a center boss (PMNBCB) for low-pressure measurements. The stresses induced in the piezoresistors and deflection of the membrane were calculated using the finite element method (FEM). The functions of the relationship between the dimension variables and mechanical performance were determined based on the curve fitting method, which can provide an approach for geometry optimization of the sensor. In addition, the values in the equations were varied to determine the optimal dimensions for the proposed membrane. Then, to further improve the sensitivity of the sensor, a series of rectangular grooves was created at the position of the piezoresistors. The proposed diaphragm was compared to existing diaphragms, and a considerable increase in the sensitivity and a considerable decrease in nonlinearity error could be achieved by using the proposed sensor. The simulation results suggest that the sensor with the PMNBCB structure obtained a high sensitivity of 34.67 mV/kPa and a low nonlinearity error of 0.23% full-scale span (FSS) for the pressure range of 0–5 kPa. The proposed sensor structure is a suitable selection for MEMS piezoresistive pressure sensors.

## 1. Introduction

Micro-electromechanical system (MEMS) piezoresistive pressure sensors are currently the most widely-used sensors in commercial and industrial applications. These sensors have considerable advantages over other sensors, such as high sensitivity, low nonlinearity error, low cost, high efficiency and small size, and they can also be easily manufactured [1,2]. These attributes are very important for the use of the piezoresistive pressure sensors in a variety of applications, such as biomedical devices [3], micro-nano manipulations [4,5], automobiles [6], aerospace technology [7] and robotics [8].

The important parts of a piezoresistive pressure sensor are the piezoresistors and the diaphragm. When pressure loading is applied to the diaphragm, stresses are generated. Piezoresistive pressure sensors operate based on the piezoresistive effect, so the piezoresistors are located on top of the diaphragm at the highest stress positions to maximize the sensor’s sensitivity [9]. During the design of the pressure sensor, the choice and optimization of new diaphragm structures are the main factors under consideration to enhance the linearity and sensitivity of the sensor. Extensive research focused on this stage of sensor development has been performed. For instance, the square diaphragm with a rectangular central boss was studied by Sandmaier and Kuhl. However, the sensitivity of this sensor was below 17.5 mV/ kPa [10]. Another sensor with the cross-beam structure placed on the membrane was introduced by Tian et al. The experimental results show that the nonlinearity error was acceptable at 0.09% full-scale span (FSS), while the sensitivity was quite low at 7.018 mV/kPa [11,12]. The structural combination of the beam and island introduced by Yu et al. showed a fairly high sensitivity of 17.795 μV/V/Pa, but the linearity error was high and the chip still large [13,14]. A peninsula structure presenting peninsulas on the flat membrane was recommended by Huang and Zhang. However, this sensor’s sensitivity of 18.4 mV/kPa was quite low for the measuring pressure of 5 kPa [15]. A peninsula island combined with a bossed diaphragm structure with a sensitivity of 0.066 mV/V/Pa and nonlinearity of 0.33% FSS was designed by Xu et al.; however, the diaphragm size was 3500 × 3500 μm${}^{2}$ [16]. In addition, a beam-membrane dual-island structure was developed by Meng and Zhao, and the sensor showed a sensitivity of 17.339 μV/V/Pa and nonlinearity of 2.559% FSS; however, the sensor’s dimensions were 7000 × 7000 μm${}^{2}$ [17]. Recently, a pressure sensor with a shuriken-structured diaphragm was also introduced by Guan et al. and showed a sensitivity of 23.6 mV/kPa and a nonlinearity of 0.18% FSS [18]. In the most recent publication, Tran et al. proposed a novel sensor chip structure based on a combination of a cross-beam, a membrane and a peninsula to make the membrane harder and increase the sensor’s sensitivity. The sensitivity was 25.7 mV/kPa for measurements in the range of 0–5 kPa [19]. Li et al. introduced a micro-pressure sensor that incorporated a four-grooved membrane combined with a rod beam to measure low pressures with a high sensitivity of 30.9 mV/V/psi, a nonlinearity of 0.21% FSS and a large chip size of 3600 × 3600 μm${}^{2}$ [20]. Yu and Huang reported a silicon membrane that was partly etched to form a crossed beam on its top for stress concentration and deposited an aluminum layer as a part of the beam. The obtained sensitivity was 0.328 mV/kPa, but the membrane dimensions were 900 × 900 μm${}^{2}$ [21]. While many diaphragm structures have been recommended in recent studies, nonlinearity and sensitivity remain important in pressure sensor design. Therefore, considerable attention has been paid to the choice of a new structure based on technical methodologies for enhancing sensor performance.

**Figure 1:**
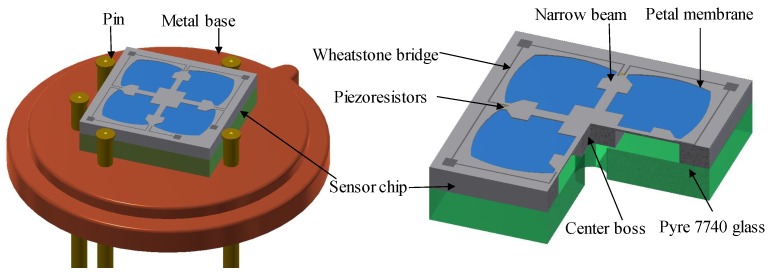
3D structure of the proposed sensor.

In this paper, a novel diaphragm structure of a piezoresistive pressure sensor with a combination of a four-petal membrane, four narrow beams and a central boss (PMNBCB) for low-pressure ranges (see Figure 1) is proposed. The finite element method (FEM) is used to estimate the stress distribution and analyze the inherent structure’s deflection for different parameters. COMSOL Multiphysics software is used in these calculations. The formulation of the proposed structure and geometry optimization are established. Then, to further improve the sensor sensitivity, a series of rectangular grooves is introduced at the location of the piezoresistors. Finally, to verify that the proposed method is optimal, the recommended sensor is compared to other sensor types.

## 2. Working Principle and Methodology

**Figure 2:**
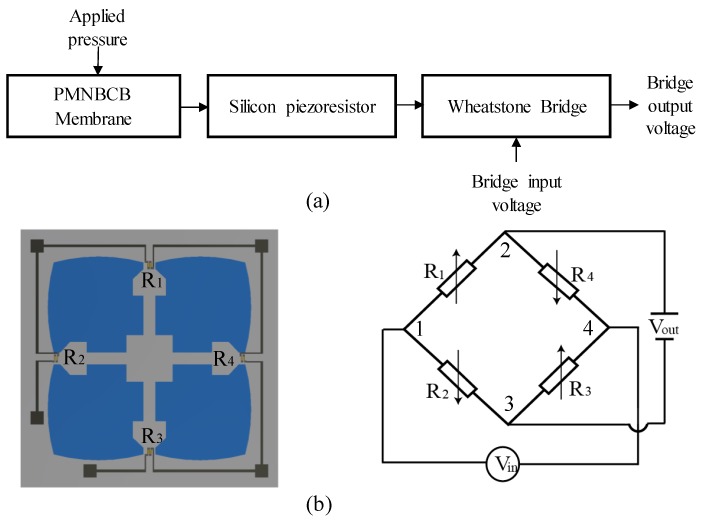
(**a**) Working principle of the piezoresistive pressure sensor; (**b**) four-petal membrane combined with narrow beams and a center boss (PMNBCB) membrane and Wheatstone bridge

Figure 2a illustrates the working principle of the piezoresistive pressure sensor. The membrane is designed to deform under applied pressure and can be fabricated using micro-machining techniques. The piezoresistors are fabricated by semiconductor technology. When pressure is applied, the sensor’s membrane will deform and induce bending stresses in the piezoresistors, which translate into a fluctuation in the resistance because of the piezoresistive effect. The Wheatstone bridge consists of silicon piezoresistors for measuring strain or displacement of the sensor element. When the Wheatstone bridge is supplying the input voltage, the variation in the resistor will lead to changes in the output voltage based on the output voltage pressure being measured.

The proposed sensor’s membrane is shown in Figure 2b, in which the four piezoresistors that comprise the Wheatstone bridge, specifically two longitudinal piezoresistors ($R_{1}$ and $R_{3}$) and two transverse piezoresistors ($R_{2}$ and $R_{4}$), are on top. In this study, the resistors are fabricated in p-type silicon with long axes in the (110) direction, and the diaphragm is designed on n-type silicon. When Nodes 1 and 4 are connected to a known input voltage $V_{in}$, an output voltage $V_{out}$ appears between Nodes 2 and 3. The value of the output voltage depends on the ratios of the resistances $R_{1}$:$R_{2}$ and $R_{3}$:$R_{4}$. When the diaphragm is subjected to pressure loading, the following stresses are experienced by the piezoresistors: the average longitudinal stress on $R_{1}$ and $R_{3}$ is $\sigma_{x1}$, and the average transverse stress is $\sigma_{y1}$; the average longitudinal stress on $R_{2}$ and $R_{4}$ is $\sigma_{x2}$, and the average transverse stress is $\sigma_{y2}$. The resistances of the piezoresistors will be changed by these stresses according to [22]:

(1) $$\begin{array}{c} \begin{array}{c} \Delta R_{1}/R_{1}=\Delta R_{3}/R_{3}=\left(\pi_{44}/2\right)\left(\sigma_{x1}-\sigma_{y1}\right)\\ \Delta R_{2}/R_{2}=\Delta R_{4}/R_{4}=\left(\pi_{44}/2\right)\left(\sigma_{x2}-\sigma_{y2}\right) \end{array} \end{array}$$

where $\pi_{44}$ is the piezoresistive coefficient with p-type silicon. The relationship between $V_{out}$ and $V_{in}$ can be given as [22,23].

(2) $$\begin{array}{c} V_{out}=V_{in}\frac{\left(\Delta R_{1}/R_{1}\right)-\left(\Delta R_{2}/R_{2}\right)}{2+\left(\Delta R_{1}/R_{1}\right)+\left(\Delta R_{2}/R_{2}\right)} \end{array}$$

Substituting Equation (1) into Equation (2), we write:

(3) $$\begin{array}{c} V_{out}=V_{in}\frac{\Delta \sigma_{xy1}-\sigma_{xy2}}{\left(4/\pi_{44}\right)+\sigma_{xy1}+\sigma_{xy2}} \end{array}$$

where $\Delta \sigma_{xy1}$ and $\Delta \sigma_{xy2}$ denote the stress difference between the longitudinal and transverse stresses, respectively ($\Delta \sigma_{xy1}=\sigma_{x1}-\sigma_{y1}$ and $\Delta \sigma_{xy2}=\sigma_{x2}-\sigma_{y2}$). In addition, the piezoresistive coefficient is a function of the doping level and temperature of p-type silicon $\pi_{44}\left(N,T\right)$, with doping level *N* at temperature *T*, expressed as follows [24]:

(4) $$\begin{array}{c} \pi_{44}\left(N,T\right)=\pi_{44}\left(N_{0},300\;K\right)P\left(N,T\right) \end{array}$$

where $\pi_{44}\left(N_{0},300\;K\right)$ is the piezoresistive coefficient at room temperature. In this study, all designs are considered under room temperature conditions (25 ${}^{{}^{\circ}}$C), and the ion implantation concentration is set to 3 × $10^{-18}$ cm${}^{-3}$. Therefore, $\pi_{44}\left(N_{0},300\;K\right)$ can be set to 138.1 × 10${}^{-11}$ Pa${}^{-1}$, and the piezoresistance factor P(N,T) is set to one. As shown in Equation (3), the sensor output depends on the different stresses, so $\Delta \sigma_{xy}$ can be regarded as a key parameter in the design of the sensor mechanical structure.

Moreover, the sensitivity is the most important parameter to evaluate the performance of the proposed pressure sensor and is determined as [22]:

(5) $$\begin{array}{c} S=\frac{\left[V_{out}\left(p_{m}\right)-V_{out}\left(p_{1}\right)\right]}{\left(p_{m}-p_{1}\right)}=\frac{V_{FS}}{\left(p_{m}-p_{1}\right)} \end{array}$$

where $p_{m}$ and $p_{1}$ are the maximum and minimum loading pressures, respectively; $V_{out}\left(p_{m}\right)$ and $V_{out}\left(p_{1}\right)$ are the measured output voltage for $p_{m}$ and $p_{1}$, respectively; and $V_{FS}$ = $V_{out}\left(p_{m}\right)$ − $V_{out}\left(p_{1}\right)$ is the full-scale output. To estimate the proposed sensor, the nonlinearity error ($NL_{i}$) is also used. This is calculated according to:

(6) $$\begin{array}{c} NL_{i}=100\%\times \left[V_{out}\left(p_{i}\right)-\frac{V_{out}\left(p_{\max}\right)}{p_{\max}}\left(p_{i}\right)\right]/V_{out}\left(p_{\max}\right) \end{array}$$

where $NL_{i}$ is the nonlinearity error and $p_{i}$ is the pressure at the tested points. $V_{out}\left(p_{max}\right)$ is the full-scale span (FSS) voltage at the maximum pressure input $p_{max}$. Hence, the nonlinearity can be either positive or negative depending on the calibration point. The maximum calculated value is called the sensor’s nonlinearity error and is normally presented in % FSS.

## 3. Sensor Design

### 3.1. Configuration Design

**Figure 3:**
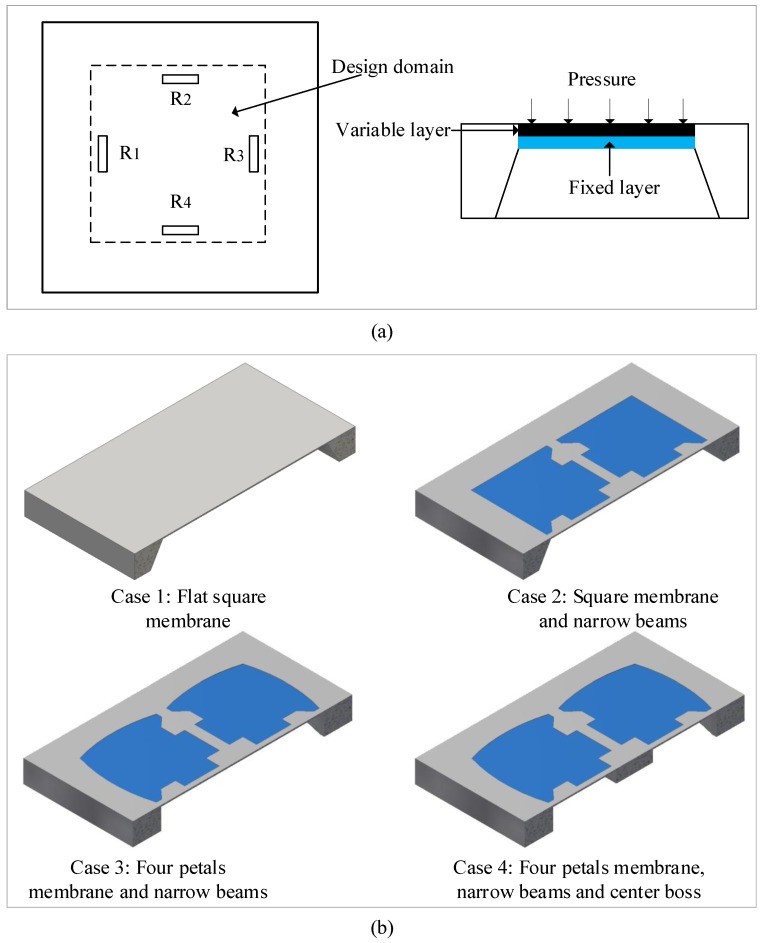
Design problem for the piezoresistive pressure sensor: (**a**) traditional flat diaphragm; (**b**) new design of the diaphragms.

The diaphragm design is the most important step among the various stages of the pressure sensor fabrication. First, we consider a traditional square diaphragm in Figure 3a. As shown, the diaphragm is fixed at all edges; pressure loading is applied to the top plane of the diaphragm; and the sensing element is a flat membrane. To improve the sensitivity and linearity, the design domain of the flat membrane is divided into two layers. The bottom layer is fixed, and the upper layer is variable. The new structure of the diaphragm is built on the fixed layer, and some materials in the variable layer are removed.

To design the proposed structure, a traditional flat square diaphragm is examined as Case 1. Then, a new sensor structure based on the combination of four narrow beams and a top square boss on the center is estimated as Case 2. Next, the square membrane is replaced by the four-petal membrane as Case 3. In the last design case, the under-center boss is added to the cavity of the sensor chip as shown in Figure 3b. These diaphragms are designed to have the same main dimensions: the feature dimensions of the sensor membranes are 2900 × 2900 μm for membrane width, 16 μm for the membrane thickness, 160 μm for the beam width and 10 μm for the beam thickness; the peninsula width and length are 500 μm; and the center boss width is 600 μm. The main objective of this work is to analyze the positioning of the membranes for the four design cases to achieve the maximum deflection sensitivity based on the small deflection theory condition (the maximum displacement of a membrane should be less than 1/5 of the membrane thickness). In all analyses, the pressure is varied from 0–5 kPa.

**Figure 4:**
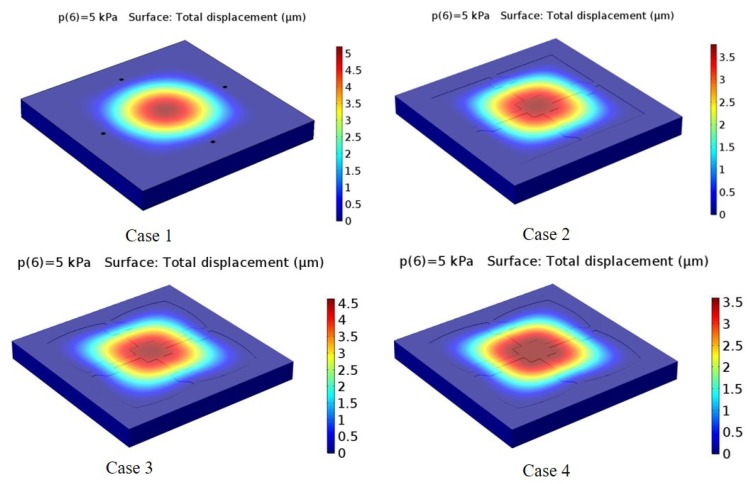
Simulated diaphragms with center deflection at 5 kPa.

The design cases of the diaphragm, along with center deflection and percentage of center deflection (compared with membrane thickness), are given in Figure 4 and Table 1 for different structures, namely Cases 1–4. The results show that the structures in Cases 1–3 yield a higher deflection sensitivity than the Case 4 structure in the range of 0–5 kPa. The maximum longitudinal stress and transverse stress are also displayed in Table 2. It is shown that the Case 3 and Case 4 structures exhibit higher stresses than the Case 1 and 2 structures. The maximum stresses of Case 3 and Case 4 are nearly equal. However, comparing both deflection and stress reveals that better performance is obtained for Case 4 than for Case 3. These results suggest that the structure of Case 4 is desirable for micro-pressure sensors in low-pressure measurement applications.

### 3.2. Mathematical Modeling

The theoretical formulas for mechanical stress and the maximal deflection for the PMNBCB structure are difficult to derive. In this study, a combination of FEM computations and the curve-fitting method is used to determine these equations approximately. The maximum deflection and the mechanical stress of the typical conventional C-type structure are shown to be the basis of the proposed structure. For a square diaphragm with clamped edges, the displacement of the diaphragm that arises from a pressure *p* can be obtained by solving the differential equation for the diaphragm. The maximum displacement of the diaphragm is obtained at the center of the membrane and is expressed as [25]:

(7) $$\begin{array}{c} w_{max}=\left[\frac{12(1-\upsilon^{2})}{47E}\right]{\left(\frac{L}{2}\right)}^{4}D^{-3}p=K_{1}L^{4}D^{-3}E^{-1}p \end{array}$$

where $K_{1}$ is a coefficient, υ is Poisson’s ratio, *E* is Young’s modulus, *L* is the membrane width and *D* is the membrane height. The maximum stresses occur at the center of the membrane edges, and the stresses are given by:

(8) $$\begin{array}{c} \left\{\begin{array}{c} \sigma_{x}=1.02{\left(\frac{L}{2}\right)}^{2}h^{-2}p=K_{2}a^{2}h^{-2}p\\ \sigma_{y}=\upsilon \sigma_{x} \end{array}\right. \end{array}$$

where $K_{2}$ is also a coefficient.

**Figure 5:**
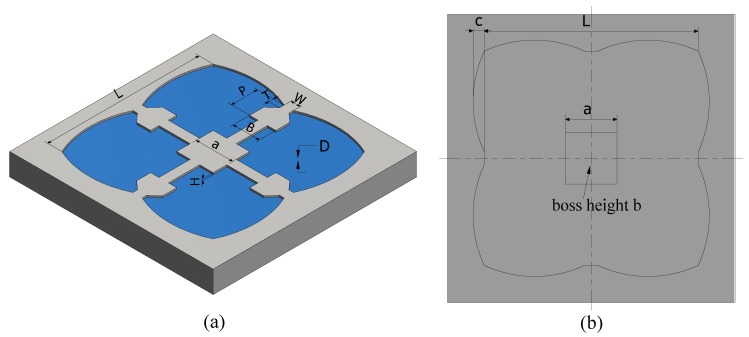
(**a**) Isometric view and (**b**) back view of the PMNBCB structure.

**Figure 6:**
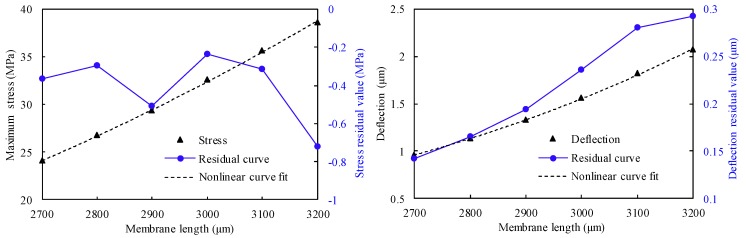
(**a**) Relationship between stress and membrane length; (**b**) relationship between deflection and membrane length

From Equations (7) and (8), it is observed that the maximum deflection and mechanical stress of the normal conventional C-type diaphragm are power functions of the structural parameters. The functional form of the proposed diaphragm can be similarly approximated in terms of power functions of each structural dimension as given by:

(9) $$\begin{array}{c} w=K_{1}\cdot L^{n1}\cdot D^{n2}\cdot H^{n3}\cdot W^{n4}\cdot B^{n5}\cdot T^{n6}\cdot P^{n7}\cdot a^{n8}\cdot b^{n9}\cdot c^{m10}\cdot p^{n11}\cdot E^{n12} \end{array}$$

(10) $$\begin{array}{c} \sigma =K_{2}\cdot L^{m1}\cdot D^{m2}\cdot H^{m3}\cdot W^{m4}\cdot B^{m5}\cdot T^{m6}\cdot P^{m7}\cdot a^{m8}\cdot b^{m9}\cdot c^{m10}\cdot p^{m11} \end{array}$$

where σ and w are the maximum longitudinal stress and maximum deflection, respectively; L,D,H,W,B,T,P,a,b and *c* are the structural dimensions, as shown in Figure 5; $K_{i}$, $n_{j}$ and $m_{j}$ (i=1,2 and j=1,2,…,11) are undetermined coefficients; *p* is the operating pressure; and *E* is the Young’s modulus. To calculate these coefficients according to Equations (9) and (10), each variable should be analyzed, whereas others are considered constant. For instance, while the impact of the membrane length *L* is examined, the value of *L* is varied in the actual range, and other variables are assumed to be constant and arbitrary. As demonstrated in Figure 6, the deflection and stress increase when the membrane length is increased. As a result, Equations (9) and (10) can be rewritten as follows:

(11) $$\begin{array}{c} w\left(L\right)=K_{1L}\cdot L^{n1} \end{array}$$

(12) $$\begin{array}{c} \sigma \left(L\right)=K_{2L}\cdot L^{m1} \end{array}$$

With the change in the membrane length, a series of *w* and σ values is obtained using the FEM simulations. Based on these simulations, the approximate power functions of σ and *w* can be derived from curve fitting using MATLAB^®^ software. The correlation between the membrane length *L* and the performance of the structure is provided in Equations (13) and (14).

(13) $$\begin{array}{c} w\left(L\right)=2\times 10^{-16}\cdot L^{4.55} \end{array}$$

(14) $$\begin{array}{c} \sigma \left(L\right)=6\times 10^{-9}\cdot L^{2.8} \end{array}$$

To confirm the goodness-of-fit between the simulation results and these equations, the residual curves of deflection and stress are also shown in Figure 6. The residual curves are defined as the differences between the actual data and the fit to the response data at each predictor value. To achieve the best goodness-of-fit, the coefficient of calculation ($R^{2}$) and residual sum of squares (RSS) are presented. The calculated results show that $R_{w}^{2}$ of Equation (13) and $R_{\sigma }^{2}$ of Equation (14) are equal to 0.9997 and 0.9993, respectively. The $RSS_{w}$ and $RSS_{\sigma }$ are equal to 0.3057 and 1.1541, respectively. These results indicate that the fitting equations and curves match the simulation results.

Similarly, the functional relations can be demonstrated for other variables. After all relationship equations are obtained, these functions are combined in Equations (9) and (10) to generate the formulas for the PMNBCB structure as follows:

(15) $$\begin{array}{c} w=K_{1}\cdot L^{4.55}\cdot D^{-0.392}\cdot H^{-0.831}\cdot W^{-0.363}\cdot B^{-0.041}\cdot T^{0.071}\cdot P^{0.0428}\cdot a^{-0.402}\cdot b^{0.0084}\cdot c^{0.0521}\cdot p\cdot E^{-1} \end{array}$$

(16) $$\begin{array}{c} \sigma =K_{2}\cdot L^{2.8}\cdot D^{-0.525}\cdot H^{-0.393}\cdot W^{-0.549}\cdot B^{0.0508}\cdot T^{0.0669}\cdot P^{-0.015}\cdot a^{-0.107}\cdot b^{0.0073}\cdot c^{0.0929}\cdot p \end{array}$$

We assume that the dimension parameters in Equations (15) and (16) are *L* = 2900, *D* = 20, *W* = 180, *H* = 14, *R* = 500, *T* = 500, *P* = 100, *B* = 500, *a* = 600, *b* = 200, *c* = 100, *p* = 5 kPa and *E* = 160 GPa. According to the simulation results, the maximum deflection is 2.38 μm, and the maximum stress is 40.70 MPa. Therefore, coefficients $K_{1}$ and $K_{2}$ can be determined after substituting these values into Equations (15) and (16). Finally, the stress and deflection equations specific to the PMNBCB structure can be presented as follows:

(17) $$\begin{array}{c} w=1.728\times 10^{-5}\cdot L^{4.55}\cdot D^{-0.392}\cdot H^{-0.831}\cdot W^{-0.363}\cdot B^{-0.041}\cdot \\ T^{0.071}\cdot P^{0.0428}\cdot a^{-0.402}\cdot b^{0.0084}\cdot c^{0.0521}\cdot p\cdot E^{-1} \end{array}$$

(18) $$\begin{array}{c} \sigma =2.465\times 10^{-10}\cdot L^{2.8}\cdot D^{-0.525}\cdot H^{-0.393}\cdot W^{-0.549}\cdot B^{0.0508}\cdot T^{0.0669}\cdot P^{-0.015}\cdot a^{-0.107}\cdot b^{0.0073}\cdot c^{0.0929}\cdot p \end{array}$$

We note that the sensor sensitivity depends on the stresses. Consequently, Equations (17) and (18) show that the membrane deflection and sensitivity are mostly dependent on the membrane width *L*, membrane thickness *D*, cross-beam height *H* and cross-beam width *W*. The dimensions of the peninsula have a weaker impact on membrane deflection and sensitivity than these parameters. On the other hand, the central boss width *a* has a significant effect on the maximum deflection of the membrane. Furthermore, as the radius of the four-petal membrane *c* increases, the stress and deformation increase, which also means that the sensitivity can be improved and that the linearity error is also increased.

### 3.3. Geometry Optimization

To determine the optimal geometrical parameters of the PMNBCB structure, we also examine the effect of each parameter on the mechanical performance. In this section, each pair of geometric parameters is examined while holding the others constant. For example, for the optimization of *H* and *D*, the values of *H* and *D* are changed, and other variables are assumed to be constant. The main objective of this work is to analyze the values of the dimensions of the structure by changing these dimensions in order to achieve the maximum deflection sensitivity within the small deflection theory conditions. The pressure range to which the sensor is subjected is varied from 0–5 kPa on the top side where the piezoresistors are to be placed. The optimized dimensions of the proposed structure, along with the membrane thickness, beam height, center deflection and percentage of center deflection, are displayed in Table 3 for different dimensions *H* and *D*, namely, C1, C2, C3, C4, C5 and C6.

The values of *H* and *D* are varied to achieve a center deflection of the proposed diaphragm less than 1/5 of the membrane thickness. The load deflection analysis shows that the diaphragms C1, C2 and C3 yield more than 20% deflection, whereas C4, C5 and C6 satisfy the permitted deflection of less than 20%. Therefore, C4, C5 and C6 satisfy the design conditions. This also shows that the membrane thickness *D* is varied between 16 and 17 μm and that the beam height *H* is varied from 12–13 μm, yielding small-scale deflection of the proposed diaphragm of dimensions 2900 μm × 2900 μm.

Similarly, the optimization of the beam width *W* and the center boss width *B* was carried out. In this case, the proposed diaphragm is created with the fixed membrane thickness and narrow beam width of 16 and 12 μm, respectively. The structure is analyzed for the deflection sensitivity at a pressure of 5 kPa for different structures, namely M1, M2, M3, M4, M5 and M6, which are presented in Table 4. The result shows a reduction in the deflection when the beam and center boss widths are increased. The maximum deflection increases to 23.94%. Due to the constraints, the maximum deflection is analyzed within 20% of the membrane thickness. The M3, M4, M5 and M6 structures satisfy the requirement for the permitted deflection of less than 20%. On the other hand, the M1 and M2 structures show greater than 20% deflection. It can also be seen that the beam width is varied between 160 and 170 μm and that the center boss width is varied from 60 μm–70 μm. Based on the micro-machining method and piezoresistor design options, the final values of the beam width and center boss width are 170 μm and 60 μm, respectively.

In the same manner, the optimization can be carried for other variables. After all of the dimensions are obtained, the PMNBCB structure’s dimension parameters are determined. The overall dimensions for the proposed diaphragm structure are summarized in Table 5.

### 3.4. Enhancement of Sensitivity

**Figure 7:**
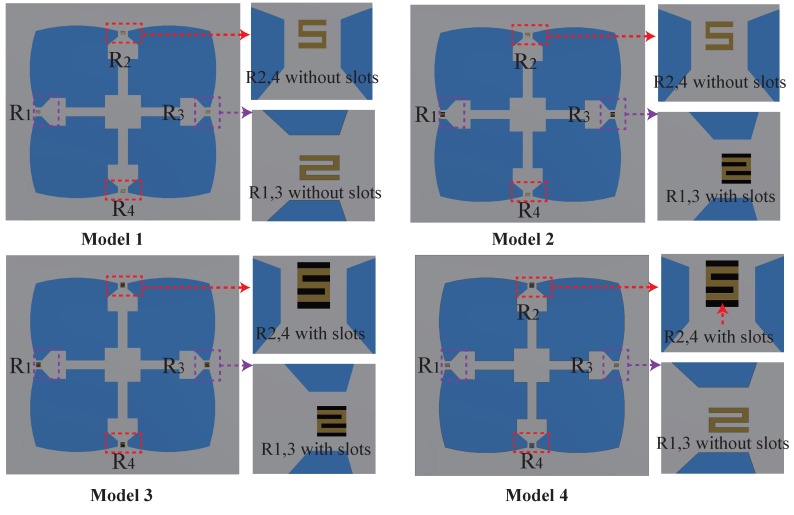
The designs of stress concentration regions (SCRs). Model 1: $R_{1,3}$ and $R_{2,4}$ without rectangular slots; Model 2: $R_{1,3}$ with rectangular slots and $R_{2,4}$ without rectangular slots; Model 3: $R_{1,3}$ and $R_{2,4}$ with rectangular slots; Model 4: $R_{1,3}$ without rectangular slots and $R_{2,4}$ with rectangular slots.

The main concept of our approach is to increase the stress in the piezoresistors’ locations. This goal is achieved through the generation of a stress concentration region (SCR) by creating holes and grooves to increase the stress. This method is highly suitable for increasing the sensitivity of the piezoresistive MEMS sensors without requiring the use of additional complicated equipment. In this study, we aim to find the SCRs to improve the sensitivity of the proposed sensor. Accordingly, a number of alternating rectangular grooves is fabricated into the piezoresistors. The geometric configurations of the proposed SCR designs are shown in Figure 7. The length and width of the slots are 12 μm and 67 μm, respectively (the same as the resistor’s dimensions). The depth of the slots is the same as the height of the narrow beam and is equal to 12 μm. Model 1 defines the proposed sensor without rectangular grooves. In Model 2, the rectangular grooves are positioned at the locations of the two longitudinal piezoresistors ($R_{1}$ and $R_{3}$). In Model 3, the rectangular grooves are generated at the location of the two longitudinal piezoresistors ($R_{1}$ and $R_{3}$) and the two transverse piezoresistors ($R_{2}$ and $R_{4}$). Model 4 defines the case where rectangular grooves are inserted only into the two transverse resistor locations ($R_{2}$ and $R_{4}$).

**Figure 8:**
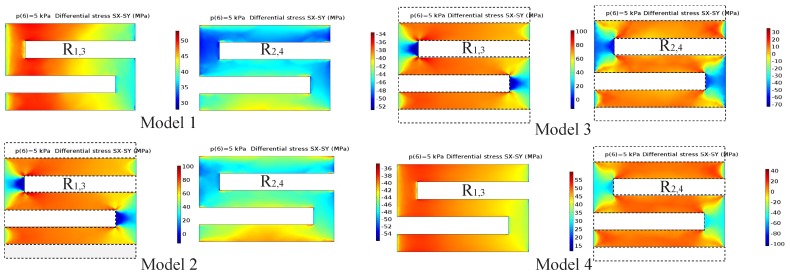
Differential stress distributions in the piezoresistors of sensor designs under a pressure loading of 5 kPa.

Figure 8 presents the numerical results for differential stress distribution induced in the piezoresistors of the four design models when subjected to 5 kPa of pressure loading on the top membrane. It is obvious that the average differential stresses increase significantly with the insertion of the rectangular slots at the resistor locations for the two longitudinal resistors. However, the average differential stress decreased rapidly when the grooves were introduced at the location of the two transverse resistors. Table 6 also shows the values of the sensor sensitivity of the model designs. Interestingly, Model 2 shows higher sensitivity. When a number of rectangular slots are inserted only into the locations of resistors $R_{1,3}$, the sensitivity of the sensor increases rapidly by 17.76%. Furthermore, when the rectangular slots are created at the locations of the four resistors, the sensitivity of the sensor decreases by 22.4%. A similar result is obtained when the slots are created at the locations of resistors $R_{2,4}$. Consequently, Model 2 is the best choice for improving the sensitivity of the piezoresistive pressure sensor.

### 3.5. Comparison to Other Sensor Structures

**Figure 9:**
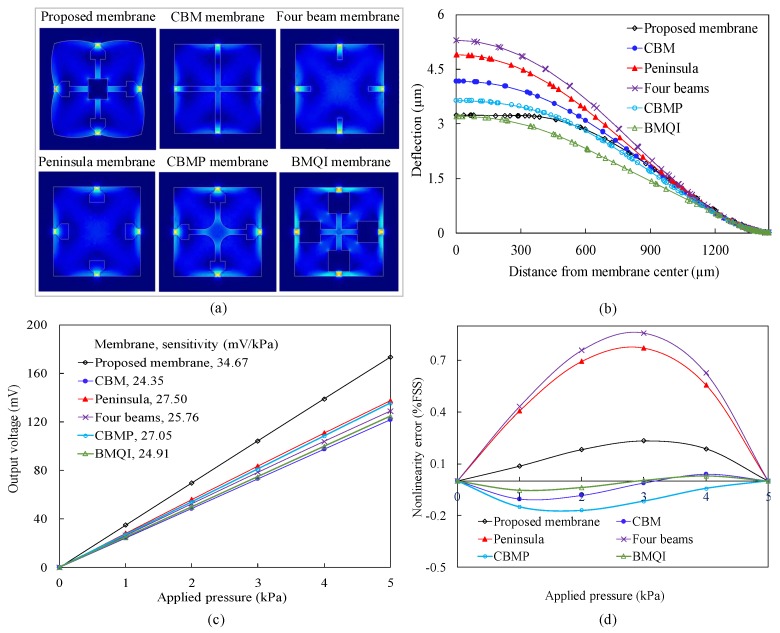
(**a**–**d**) Comparison to other structural membranes.

The optimized PMNBCB structure proposed in this work (see Figure 9a) is compared to the CBM structure [11,12], the peninsula structure [15], the four-beam structure [20], the CBMP structure [19] and the BMQI structure [14]. These membranes are redesigned to have equal prime dimensions. For example, sensor membranes show dimensions of 2900 μm × 2900 μm for membrane width, 16 μm for membrane thickness, 170 μm for beam width and 12 μm for beam thickness. Figure 9b–d demonstrates the calculated performance of these sensors under loading pressures of 0–5 kPa with an input voltage of 5 V DC.

Examination of the overall results of the analysis shows that the proposed sensor structure provides the best sensor performance in terms of sensitivity, deformation and nonlinearity error. Compared to two high-sensitivity sensors, namely the peninsula and CBMP membrane, the proposed sensor exhibits a nearly 26% increase in sensitivity and approximately 35% and 11% decreases in maximum deflection, respectively. Furthermore, the nonlinearity error of the proposed sensor is only approximately 0.23% FSS. These results show that the PMNBCB structure produces higher sensitivity than the CBMP membrane and peninsula membrane. After a square membrane is replaced with the four-petal membrane and the under boss is added to the center of the membrane, the deflection and sensitivity of the proposed sensor improve considerably. Compared to the BMQI sensor, the proposed sensor shows the same amount of membrane deflection; however, the sensitivity of BMQI is lower (24.91 mV/kPa) than the sensitivity (34.67 mV/kPa) of the proposed sensor. The analysis also finds that compared to the PMNBCB sensor, the CBM sensor has low sensitivity, high nonlinearity error and large displacement. Finally, the analysis indicates that the special four-beam membrane has a very large displacement of up to 5.3 μm under an applied pressure of 5 kPa, while the deflection of the proposed membrane is only 3.2 μm. In addition, the sensitivity of BMQI is not as high at 24.91 mV/kPa, and the nonlinearity error is high at 0.8% FSS.

During the design process of a piezoresistive pressure sensor, the change in the membrane structure and dimensions can result in the gain in sensitivity and the increase of the mechanical non-linearity. The proposed PMNBCB structure has been developed to overcome this limitation and to improve the sensitivity and linearity. First, the combination of four narrow beams and the central island with the flat membrane significantly increased the stress at the location of the resistor while also reducing the deformation. Thus, the sensitivity of the senor is increased, and its nonlinearity error is reduced. Then, the square membrane is converted into a four-petal membrane, which increases both the sensitivity and the nonlinearity error. To overcome this situation, a central boss is added at the bottom of the membrane. Finally, a number of alternating rectangular grooves is fabricated into the piezoresistors, resulting in improved sensor sensitivity. These results clearly show that the PMNBCB structure is indeed a high-performance structure and is suitable for micro-pressure sensors in MEMS applications.

## 4. Fabrication Process

**Figure 10:**
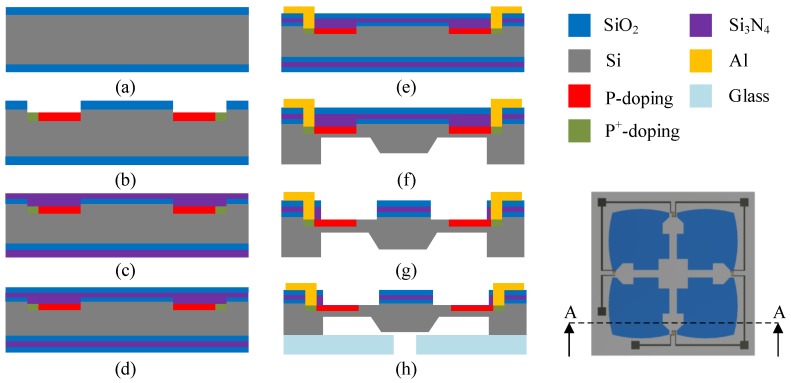
(**a**–**h**) Main fabrication process of the proposed sensor chip.

The bulk micro-machining technology is employed to fabricate the proposed sensor chip from a standard 400 μm-thick n-type (100) silicon wafer. Photolithography is applied, and six masks are used to form the metal layer and sensing elements. Boron implantation is used to pattern the p-type piezoresistors. The main steps in the fabrication process are illustrated in Figure 10. (1) In the first step, thermal oxidation is applied to produce the thin layers of SiO${}_{2}$ on both surfaces of the wafer. These oxide layers are used to separate the metallization from the silicon substrate Figure 10a. (2) After the thermal oxidation process, lithography and ion implantation of boron are performed. Therefore, the p-type piezoresistors and heavily-doped contact regions are patterned and formed on the front side of the silicon wafer (Figure 10b). (3) Subsequently, low-pressure chemical vapor deposition (LPCVD) is adopted to grow the passivation layers of Si${}_{3}$N${}_{4}$ to protect the piezoresistors (Figure 10c). (4) Then, thin films of SiO${}_{2}$ are deposited again via the LPCVD process to act as electrical insulation (Figure 10d). (5) After that, the reactive ion etching (RIE) process is used. The thin aluminum layer is deposited and patterned on the top of the sensor chip to create the electrical connection areas between the piezoresistors and lines of the Wheatstone bridge (Figure 10e). (6) In the next step, the KOH wet etching process is used on the back side of the silicon wafer to form a deep cavity (Figure 10f). (7) To obtain the PMNBCB structure and SCR, the RIE process is applied again to produce the four-petal membrane, narrow beams and rectangular slots on the front side of the silicon wafer (Figure 10g). (8) In the final step, the bottom side of the sensor chip is attached to Pyrex 7740 glass by the anodic bonding process (Figure 10h).

## 5. Conclusions

In this work, to optimize linearity and sensitivity, a novel PMNBCB structure of a silicon piezoresistive pressure sensor is proposed. FEM simulations were used to predict the stresses induced in the piezoresistors owing to membrane deflection under different pressures. Based on the curve-fitting method, the equations for the relationship between the dimension variables and mechanical performance were determined and provide guidance for the design of the sensor with the PMNBCB structure. Varying the values in these equations allowed the optimal geometry of the proposed structure to be determined. Then, to further improve the sensitivity of the sensor, a series of rectangular grooves was created at the positions of the resistors. As a result, the sensitivity of the sensor is significantly increased. To illustrate the optimization of the proposed sensor, the sensor is evaluated based on the analysis of and comparison with the CBMP, CBM, peninsula, BMQI and four-beam structures. The calculation results suggest that the proposed structure not only increases the sensor sensitivity, but also reduces the nonlinearity error. Finally, the main fabrication processes of the proposed sensor chip based on bulk micro-machining and anodic bonding technology are presented. These results indicate that the PMNBCB structure is suitable for piezoresistive pressure sensors in MEMS applications.

## Figures and Tables

**Table 1:** Pressure versus center deflection for the four design cases.

Pressure (kPa)	Case 1	Case 2	Case 3	Case 4
0	0.00	0.00	0.00	0.00
1.00	1.08	0.76	0.94	0.73
2.00	2.14	1.52	1.88	1.46
3.00	3.19	2.28	2.82	2.19
4.00	4.22	3.04	3.74	2.90
5.00	5.21	3.79	4.64	3.50
% deflection	32.54	23.67	29.00	21.88

**Table 2:** Comparison of the maximum longitudinal stress and transverse stress.

Membrane	Longitudinal Stress (MPa)	Transverse Stress (MPa)
Case 1	28.566	7.0979
Case 2	45.309	9.0385
Case 3	63.042	15.05
Case 4	58.628	13.726

**Table 3:** Pressure versus center deflection for diaphragms with *D* and *H*.

Pressure Mpa	C1 *D* = 14 *H* = 10	C2 *D* = 15 *H* = 11	C3 *D* = 16 *H* = 12	C4 *D* = 17 *H* = 13	C5 *D* = 18 *H* = 14	C6 *D* = 19 *H* = 15
1	0.98	0.70	0.76	0.55	0.46	0.39
2	1.94	1.40	1.52	1.10	0.93	0.79
3	2.89	2.09	2.28	1.64	1.39	1.18
4	3.81	2.78	3.04	2.18	1.84	1.57
5	4.71	3.45	3.79	2.72	2.30	1.96
% deflection	33.61	22.99	23.68	16.00	12.78	10.31

**Table 4:** Pressure versus center deflection for diaphragms with *W* and *B*.

Pressure MPa	M1 *W* = 140 *B* = 40	M2 *W* = 150 *B* = 50	M3 *W* = 160 *B* = 60	M4 *W* = 170 *B* = 70	M5 *W* = 180 *B* = 80	M6 *W* = 190 *B* = 90
0	0.00	0.00	0.00	0.00	0.00	0.00
1	0.78	0.71	0.66	0.59	0.54	0.49
2	1.55	1.42	1.31	1.18	1.07	0.97
3	2.32	2.12	1.96	1.76	1.61	1.45
4	3.08	2.81	2.61	2.34	2.14	1.93
5	3.83	3.49	3.24	2.91	2.66	2.41
% deflection	23.94	21.84	20.26	18.19	16.63	15.03

**Table 5:** Optimized dimensions of the proposed sensor.

Parameter	*L*	*D*	*W*	*H*	*B*	*T*	*P*	*a*	*b*	*c*
Dimension	2900	16	170	12	500	500	100	650	200	100

**Table 6:** Comparison of differential stress and sensitivity for four design cases.

Design	Average ($\sigma_{x1}$ − $\sigma_{y1}$)	Average ($\sigma_{x2}$ − $\sigma_{y2}$)	Sensitivity
Model 1	42.9	−42.39	29.44
Model 2	63.92	−45.63	34.67
Model 3	65.09	−2.48	22.83
Model 4	46.28	−1.59	16.28
